# Discovery of Urinary Biomarkers of Seaweed Intake Using Untargeted LC–MS Metabolomics in a Three-Way Cross-Over Human Study

**DOI:** 10.3390/metabo11010011

**Published:** 2020-12-28

**Authors:** Muyao Xi, Lars Ove Dragsted, Mikkel Tullin, Madeleine Ernst, Nazikussabah Zaharudin, Giorgia La Barbera

**Affiliations:** 1 Department of Nutrition, Exercise, and Sports, University of Copenhagen, 1958 Copenhagen, Denmark; muyao@nexs.ku.dk (M.X.); mikkel.tullin@iqvia.com (M.T.); nazikussabah@ump.edu.my (N.Z.); glb@nexs.ku.dk (G.L.B.); 2 Section for Clinical Mass Spectrometry, Danish Center for Neonatal Screening, Department of Congenital Disorders, Statens Serum Institut, 2300 Copenhagen, Denmark; maet@ssi.dk; 3 Faculty of Industrial Sciences and Technology, Universiti Malaysia Pahang, Gambang 26600, Malaysia

**Keywords:** seaweed, biomarkers of intake, untargeted metabolomics

## Abstract

Seaweeds are a marine source rich in potentially bioactive components, and therefore have attracted attention since the middle of the twentieth century. Accurate and objective assessment of the intake of seaweeds to study their health effects is hampered by a lack of validated intake biomarkers. In this three-armed, randomized, cross-over study, an untargeted metabolomics approach was applied for discovering novel intake biomarkers. Twenty healthy participants (9 men and 11 women) were provided each of three test meals in a randomized order: 5 g of *Laminaria digitate* (LD), 5 g of *Undaria pinnatifida* (UP), or a control meal with energy-adjusted pea protein. Four urine samples and a 24 h pooled urine were collected along with blood samples at seven time-points. All samples were profiled by LC-ESI-QTOF-MS and the data were analyzed by univariate analysis and excretion kinetics to select putative intake biomarkers. In total, four intake biomarkers were selected from urine samples. They were identified as hydroxyl-dihydrocoumarin at Level III, loliolid glucuronide at level I, and isololiolid glucuronide at level II, while the last one remains unknown. Further identification and validation of these biomarkers by a cross-sectional study is essential to assess their specificity and robustness.

## 1. Introduction

Seaweed is classified into three types based on its characteristic pigmentation, i.e., brown, red, and green macroalgae [1]; the brown species are thought to contain mixed pigments from both of the other two classes, with fucoxanthin as an important bioactive xanthophyll pigment [2]. Seaweed, as a traditional food product in Asian countries, is increasingly attracting attention due to its content of potentially bioactive compounds [1,3,4], including phenolic acids [5], phlorotannins [6], terpenoids [7], and certain unsaturated fatty acids [8]. Seaweed might potentially reduce the risks of obesity, hypertension, and even cancer, but so far the evidence is insufficient and human trials are needed [9,10,11,12]. However, providing real evidence for the health effects of seaweed in human studies is quite difficult, especially due to the difficulties in quantitative intake assessment [13]. Therefore, some objective and sensitive tools are essential for confirming how much seaweed is consumed, especially because the amounts consumed may often be low.

Subjective reporting, such as food frequency questionnaires (FFQ) or 24 h recalls (R24h), are currently used in most human studies for the assessment of food intake, but they lack precision and accuracy [14]. In order to establish an objective and accurate dietary assessment tool, biomarkers of food intake (BFIs) might instead be applied for both monitoring compliance in the randomized controlled trials (RCTs) [15,16] and supporting intake assessments in observational studies. Untargeted metabolomics facilitates the discovery of novel BFIs [17] because it allows the measurement of unknown food-related metabolites in biofluids [18,19] with powerful analytical techniques such as ultra-high performance liquid chromatography (UHPLC) coupled to high-resolution mass spectrometry (HRMS). The metabolic profile is composed of hundreds of features that are recognized by their measured mass to charge ratio (*m/z*), retention time (RT), and relative abundance. The combination of mass spectrometry with chemometrics is efficient for analyzing metabolomics data to obtain BFIs [20]. Validation of selected BFIs is crucial before they can be further applied, including plausibility, dose/time-response after a single exposure or multiple exposures, robustness, reliability, stability, analytical performance, and reproducibility [21].

The three-way cross-over study design underlying this paper was designed 1) to investigate the influence of *Laminaria digitate* (LD) and *Undaria pinnatifida* (UP) on postprandial glucose and insulin responses [22] and 2) to explore the blood and urinary metabolomes after seaweed consumption. In the present study, we aimed at the second objective to discover and identify seaweed intake biomarkers in urine or blood using a data-driven metabolomics approach. This will support future studies on the effects of brown seaweeds on human health. 

## 2. Results

The whole procedure of managing participants followed the CONSORT 2010 flow diagram. The contents reported throughout the paper were addressed according to each item demonstrated in both the CONSORT 2010 statement and Adaptive designs CONSORT Extension (ACE) statement [23,24]. 

### 2.1. Data Analysis

#### 2.1.1. Data Preprocessing

Forty subjects were screened and twenty healthy subjects (nine men and eleven women, aged 28.8 ± 5.4 years) were enrolled in this study. No one dropped out. 

In total, 420 blood samples (three meals × seven time-points × 20 participants), 300 urine samples (three meals × five time-points × 20 participants) were collected from participants and injected into LC-ESI-QTOF-MS. The raw data were preprocessed by MZmine2 and cleaned by algorithms in MATLAB to remove very early and late eluting peaks as well as implausible masses. After data cleaning, 3196 features in positive mode and 2337 features in the negative mode were selected for urine, and 899 in positive mode and 437 in the negative mode for blood.

#### 2.1.2. Univariate Analysis

The n-way ANOVA gave an overview of which features differed according to the meals and time-points. The Dunn–Šidák multiple comparison tests were applied to further filter out irrelevant features. In conclusion, a total of 14 (positive mode) and 6 (negative mode) features were selected. Visual inspection of the excretion curves of the 20 features was applied, and eventually four (positive mode) and three (negative mode) features (Supplementary Material S1) were selected because they showed plausible kinetics and significant differences between time-points (*p* < 0.04) in the seaweed meals. These are listed in Table 1 with *m/z*, RT, and the observed fragments from each precursor. Note that one feature (*m/z* 137.0596, RT 4.63) only differed for UP treatment and not for LD treatment compared with the control. The seven significant features were also verified in 24 h pooled urine samples (Supplementary Material S1.1, sixth column). The blood could not provide us with any information because the quality of the data was poor. 

### 2.2. Identification of Putative Intake Biomarkers of Seaweed

Among the seven selected significant features in both modes, there were four compounds selected as putative intake biomarkers as shown in Table 1, M1–M4.

**Figure 1:**
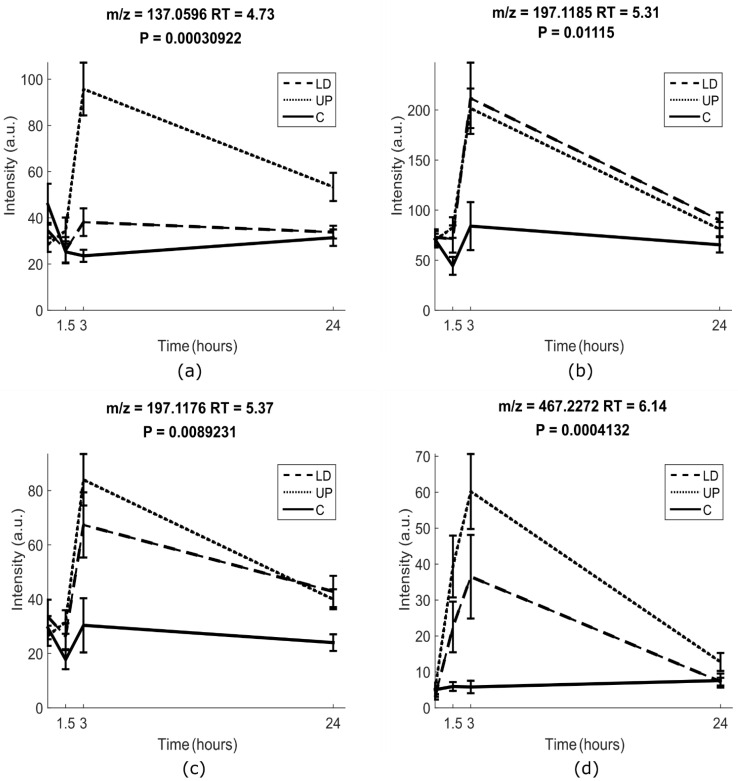
Urine excretion profiles of four new candidate BFIs for seaweed intake (**a**), M1; (**b**), M2; (**c**), M3; (**d**), M4; see text), showing the excretion profile in time (hours) against biomarker intensity, i.e., the average peak area at each time point, with error bars and *p*-values for the meal factor. Each treatment is shown in the legend with different line styles: LD, *Laminaria digitata*, UP, *Undaria pinnatifida*; C, control.

The metabolite M1 could be tentatively identified as hydroxyl-dihydrocoumarin (GPJCOQPUQUEBTB-UHFFFAOYSA-N) by MS and MS/MS spectra after manual inspection, because the spectral pattern was much more similar to that of 7-hydroxycoumarin after comparing with 4-hydroxycoumarin (Supplementary Material S2.1a,b). Indeed, the presence of a peak at *m/z* 165.0540 at the same retention time, suggested that this *m/z* represented the potential precursor, while the peak at *m/z* 137.0596 was an in-source fragment, after a loss of –CO (Supplementary Material S2.1a,b upper images). The MS/MS fragmentation pattern of *m/z* 165.0540 included and overlapped with that for *m/z* 137.0596 (Supplementary Material S3). M1 was therefore tentatively identified as hydroxyl-dihydrocoumarin, at level III identification. M1 was significantly different only between the UP treatment and the control (Figure 1a), indicating that M1 might be specific for the consumption of UP seaweed.

**Figure 2:**
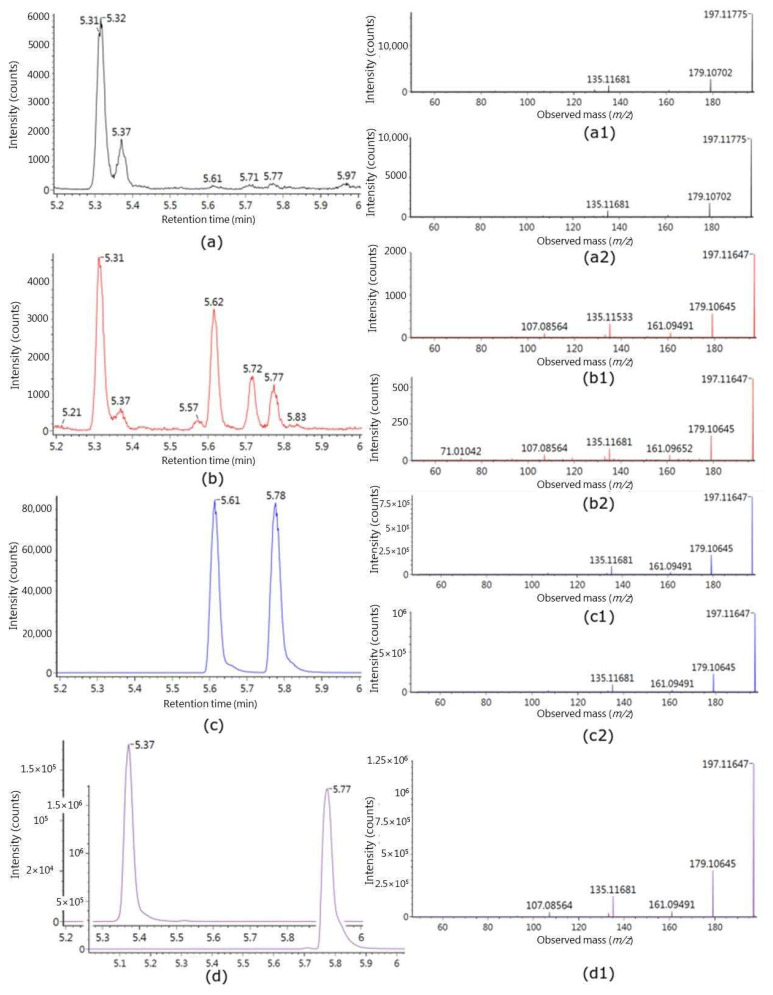
In the left panel: comparison of the extracted ion chromatograms of *m/z* 197.1185 (mass tolerance < 5 ppm) in full-scan MS of: (**a**) normal urine sample; (**b**) glucuronidase treated urine sample; (**c**) seaweed extract; (**d**) loliolid commercial standard (in the front) and synthesized loliolid glucuronide from the loliolid commercial standard (in the back). The chromatographic peaks of M2 and M3, namely loliolid glucuronide and isololiolide glucuronide, are at RT 5.31 and 5.37, respectively. The chromatographic peaks of the deconjugated (glucuronidase treated) M2 and M3, namely loliolid and isololiolide, are at RT 5.61 and 5.78, respectively. In the right panel: comparison of the MS/MS spectra of the *m/z* 197.1185 shown in the relative extracted ion chromatograms, in particular: in (a1) and (a2) MS/MS of the peak at RT 5.31 and 5.37, respectively; in (b1) and (b2) MS/MS of the peak at RT 5.62 and 5.77, respectively; in (c1) and (c2) MS/MS of the peak at RT 5.61 and 5.78, respectively; in (d1) MS/MS of the peak at RT 5.77. The collision energy used for all MS/MS was 14 eV.

The metabolites M2 and M3 distinguished both LD and UP treatment from the control, as shown in Figure 1b,c. M2 (HSUGIWKEPZWUFB-UYLUDEDWSA-N) and M3 (HSUGIWKEPZWUFB-VKMZOEIRSA-N) were identified as the glucuronide conjugate of loliolid and (tentatively) that of isololiolid, respectively. Indeed, the MS/MS spectrum of the core compound of M3 (peak at *m/z* 197.1176 in positive mode, obtained after the loss of glucuronic acid from the precursor in MS) was matched with the compound, loliolid, in the GNPS library (Supplementary Material S4). The match was classified in the GNPS platform as a bronze level with the cosine value 0.76 with seven peaks from the MS/MS spectrum of 197.1176 matching with the MS/MS spectrum of loliolid. We report here that M3 also matched with a synthesized loliolid glucuronide in terms of RT, *m/z*, and fragmentation pattern. Therefore, M3 was identified at level I as loliolid glucuronide. The MS/MS spectral pattern of M2 was quite similar to that of M3. Moreover, the similarity with respect to the RTs of the two metabolites suggested that they could be diastereoisomers. Therefore, M2 was identified as isololiolid glucuronide at level II. Further information about M2 and M3 could be obtained by comparison of the seaweed extracts and the deconjugated urine samples. After urine deconjugation with glucuronidase, two peaks appeared at *m/z* 197.1176 in positive mode, at the same RTs and with the same *m/z* as two peaks detected in the seaweed extract. Additionally, the fragmentation patterns of the peaks in the deconjugated (glucuronidase treated) urine and in the seaweed extract matched with those of M2 and M3 after glucuronic acid loss (Figure 2).

Finally, M4 also distinguished both LP and UP meals from the control meal as shown in Figure 1d. However, the MS/MS spectrum of M4 did not suggest any specific structure, aside from a multitude of fragments typical of sugars. Therefore, M4 may be either a sugar conjugated compound, a complex carbohydrate, or a polyhydroxylated aliphatic compound.

## 3. Discussion

The current work aimed to discover BFIs associated with seaweed consumption. A combination of univariate data analysis and inspection of the excretion curves was applied to screen and obtain the final four putative BFIs. All of the putative BFIs were discovered from urine, and none in blood. Metabolite M1 distinguished UP consumption from LD as well as the control, and this metabolite may therefore be specific for *Undaria pinnatifida*, but further research is needed to investigate its specificity and to fully elucidate its structure. The other three observed compounds, M2–M4, were found in the urine after consumption of both types of brown seaweed, indicating that they could be BFIs of brown seaweed consumption in general or possibly even for a broader range of seaweeds; their specificity will therefore also await further study such as a broader screening of loliolids across seaweeds. 

M1 was tentatively identified as hydroxyl-dihydrocoumarin at level III. Indeed, its fragmentation pattern strongly resembles the MS/MS spectrum of 7-hydroxycoumarin after comparing M1 with 7-hydroxycoumarin and 4-hydroxycoumarin reported in the mzCloud library, as shown in Supplementary Material S2.1. Meanwhile, SIRIUS predicted that it belongs to the benzenediols, in which additional hydroxyl groups are attached to the benzene ring [25]. SIRIUS also pointed out that M1 could be hydroxyl-dihydrocoumarin based on the MS/MS mass spectrum of M1. However, there is no unequivocal candidate structure. Firstly, the relative position of the functional groups is uncertain, especially the position of the hydroxy group [26]. Secondly, we cannot exclude that the compound is an in-source fragment of a larger metabolite, due to its late elution time (4.63 min) and the absence of any adduct ions (sodium, potassium, or dimers) confirming that either *m/z* 137.0596 or *m/z* 165.0547 are the protonated adducts [M + H] of the parent ion. Additionally, in the seaweed extracts, both 165.0540 and 137.0524 masses were present at RT 1.63 min (data not shown), suggesting that both ions could represent fragments from an original larger compound found in seaweed. Therefore, further investigation is needed for ascertaining the final structural identification. However, the hypothesis that the compound could belong to the class of coumarins is supported by the fact that coumarins have been already found in some marine macroalgae [27,28]. The excretion profile of M1 (Figure 1a) demonstrates that it is unique to UP and not excreted after LD. According to the validation criteria [21], this means that M1 is plausible (present in UP extract) and with an appropriate time-response (i.e., excretion kinetics) after a single exposure. However, work is still needed to show that, as a BFI, it is reproducible, robust, has good (targeted) analytical performance, and that it is stable during the storage of urine samples or seaweed. These criteria need to be validated in a larger cohort study and experimentally in human dose–response studies with the seaweeds. Besides, it should be investigated whether M1 is specific to UP or a certain subgroup of brown seaweeds as already discussed.

M2 and M3 (both with the theoretical monoisotopic mass, *m/z* 372.1419) were found to be phase II metabolites resulting from glucuronidation [29]. The unconjugated compounds (monoisotopic mass 196.1176) originate directly from seaweed, as shown by the comparison of the seaweed extract and the glucuronidase-treated urine (Figure 2b(b1, b2),c(c1,c2)). Here, two new peaks at *m/z* 197.1176, which were absent in the original urine samples, appeared at the same RT (RT = 5.62, 5.77) as those in the seaweed extract. This demonstrates that the compounds were originally present in seaweed, but after absorption, they were conjugated with glucuronic acid and excreted into the urine. The unconjugated form of M3 was annotated by GNPS [30] as loliolid, with a cosine score of 0.76, with most MS/MS fragments matching those from loliolid (Supplementary Material S4). The commercial standard also confirmed that M3 was loliolid (Figure 2a–d, a2–d1, Supplementary Material S2.2c1,c2) in terms of RT, *m/z*, and fragmentation pattern. The spectral pattern of M2 was almost identical with that of M3, and the RTs of the two compounds were very similar, suggesting that M2 may be a diastereoisomer of M3, i.e., isololiolid glucuronide (Figure 2a, a1 vs a2). The comparisons of MS/MS of the commercial standard, M3, and M2 are shown in Supplementary Material S2.2a,b. Others [31,32,33,34] have reported that both loliolid and isololiolid can be isolated from seaweeds. The compound has been shown to have bioactivity in model systems, potentially protecting the seaweeds [35,36]. Loliolid has been reported to be a degradation product of certain carotenoids [36] and has also been reported to be present in a South African scrub used in folk medicine [37]. Therefore, the specificity as BFIs for brown seaweeds or seaweeds, in general, will depend on the presence of loliolid in other foods, i.e., the robustness of these two markers should be confirmed in an observational setting among seaweed consumers and non-consumers. According to BFI validation criteria [21], M2 and M3 are plausible, have an appropriate time-response based on the excretion curve, and the 24 h urine samples, and commercial standards allow us to potentially measure them quantitatively. However, the robustness and storage stability of M2 and M3 are still unknown, and a larger cross-sectional study and an experimental dose–response study are needed for further validation of loliolid(s) as BFIs for edible seaweeds. Additional studies of other edible seaweeds would also help to indicate the potential specificity of these markers.

M4 (*m/z* 467.2272) was measured in the negative mode and some smaller fragments in the MS/MS fragmentation of M4 demonstrated that it has a substructure similar to that of glucuronic acid (Supplementary Material S2.3a,b). However, neither the fragment corresponding to the unconjugated metabolite nor other informative fragments could be observed in the spectral pattern (Supplementary Material S2.3c). In order to check whether M4 was a glucuronic conjugate, a urine sample with a high abundance of M4 was deconjugated. After deconjugation, the intensity of M4 only slightly decreased, and no peak corresponding to the unconjugated metabolite appeared. Moreover, M4 was not observed in the seaweed extract profiles. Therefore, we hypothesize that M4 derives from a glycoside or carbohydrate present in the seaweeds (Supplementary Material S2.3a,b); M4 is most likely resulting from humans rather than microbial biotransformation, as seen from the fast excretion profile (Figure 1d).

Although the metabolites M1–M3 seem to be promising biomarkers of seaweed intake, the intensity of the baseline of the excretion curves of M1–M3 (Figure 1a–c) is not zero, indicating that these compounds may not be specific to brown seaweed and might also be present in other plants [36] at much lower levels. Using combined biomarkers could be a good solution to address the potential lack of specificity of individual biomarkers for particular foods, as reported previously [21,38]. In one review [38], hydroxytrifuhalol A, 7-hydroxyeckol, C-O-C dimer of phloroglucinol, diphloroethol, fucophloroethol, dioxinodehydroeckol, and/or their glucuronides or sulfate esters, as well as fucoxanthinol were all considered as candidate intake biomarkers for brown seaweed. However, none of these were discovered to be significant BFIs, either from urine or blood in this controlled cross-over study. We assume the reasons are: (1) the instrumentation and method used for measuring seaweed compounds differ between previous studies and the present one; (2) the intake of 5 g of seaweed in this study design may not have been enough to make the candidate BFIs detectable in biofluid samples; (3) the matrix from either seaweed itself or from biofluids affected the discovery of the candidate BFIs. Our candidate BFIs discovered in this study were not mentioned in the review because they had not previously been detected in human samples after seaweed intake. However, these seaweed BFIs look particularly promising due to the sensitivity after low doses, and main gain specificity if used in combination. Further validation of the specificity of combined biomarkers will enhance the provision of qualitative biomarkers to predict whether brown seaweed was indeed ingested.

## 4. Materials and Methods 

### 4.1. Subjects

Twenty healthy subjects (aged 28.8 ± 5.4, BMI = 21.4 ± 2.1 kg/m^2^) were recruited via posters at the University of Copenhagen and by advertisement on websites (http://www.forsogsperson.dk and www.sundhed.dk) as detailed previously [22]. Participants who met the following criteria were excluded: (1) systemic infections; (2) acute or chronic metabolic disease; (3) smokers or drug addicts; (4) pregnant, planning pregnancy or breastfeeding; (5) iodine-associated intolerance or allergy; (6) surgery for obesity; (7) assigned for any human intervention study in the past four weeks before starting this study; (8) habitual alcohol intake above the recommended maximal limit by the Danish Health and Medicines Authority (7 and 14 drinks per week for women and men, respectively). All participants who were screened over the phone were invited for an information meeting and were then recruited if they agreed to participate in the study. All study participants provided written consent. The study was registered at Clinicaltrials.gov (ID# NCT02608372).

### 4.2. Study Design

**Figure 3:**
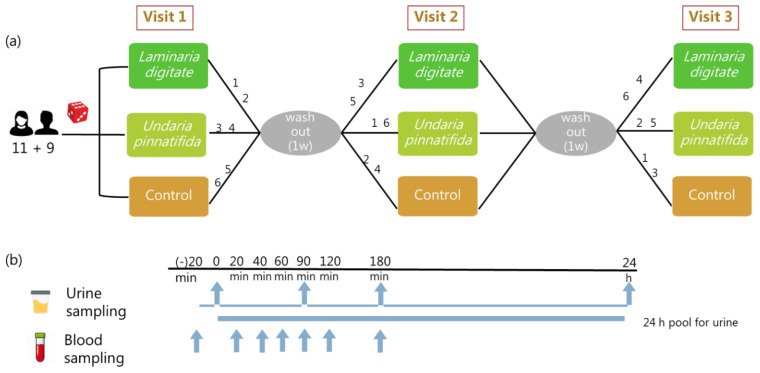
Three-way cross over meal study design (**a**) in which three meals were provided in random order at three visits, separated by one-week washout intervals. The numbers on the arms represent the six possible sequences of the meal interventions. Biofluid collections (**b**) were performed as shown by the blue arrows, for each test day.

The study design was authorized by the Ethical Committee of the Copenhagen region (journal no.: H 15004500) in accordance with the Helsinki-II declaration, and data safety procedures were authorized by the data inspection authority at Copenhagen University. The study was carried out at the Department of Nutrition, Exercise, and Sports (NEXS), University of Copenhagen (KU) starting from May 2015 until August 2015. The study had a randomized, 3-way, semi-blinded cross-over design consisting of three test meals including LP, UP, and a control meal. The qualified participants were randomized into six groups according to the six possible sequences of the meal interventions, generated by the RAND function in Microsoft Excel. The order in which the subjects consumed the test meals is represented by the numbers on the arms of the study design in Figure 3a. The test meals were served on the test day of each period, and the wash-out period was at least seven days between meals. The participants were not informed about which meal they received, but it was impossible to fully eliminate seaweed taste and, consequently, it was a semi-blinded study design; however, the participants were unlikely to discriminate between the two seaweeds, LD and UP. Participants were instructed to avoid all species of seaweed, as well as intake of paracetamol, 48 h before and throughout each test day, apart from what was provided in the study. Furthermore, participants were asked to refrain from any caffeinated beverages, such as coffee, tea, cola, energy drinks, and chocolates, as well as alcohol during the same period. Besides, intense physical activity was not to be performed from 24 h before each test day and until the following morning. Participants were fasting from 20:00 before each test day until 08:00 the next day, but 0.5 L of water was consumed during this period. 

### 4.3. Test Meals

Dried LD (5 g, AlgAran Teoranta, Lilcar Co. Donegal, Ireland), dried UP (5 g, JFC Deutschland, Dusseldorf, Germany), or 83% pea protein (5 g, Natur Drogeriet, Hørning, Denmark) were each provided as one of three test meals by the kitchen staff at NEXS, KU at 08:45 of each test day. Together with each test meal, 150 mL drink containing 30 g corn starch and 22 g sugar-free lemonade powder (Fun One, Stevia lemonade with guava/lime from Kavli A/S, Hvidovre, Denmark) were served as well. In order to preserve its constituents, seaweed processing was limited. The two types of dried seaweed (LD and UP) were immersed in 200 mL water until sufficiently soft (~15 min). They were then rinsed and drained from excess water. The soft seaweed preparations were finally minced into small pieces, mixed with 0.5 g iodized table salt, 0.2 g black pepper, and 4 g fresh lemon juice to improve the palatability. This last step was also performed for the water-soaked pea protein control meal. Water (0.5 L) was additionally served with each meal. The participants received an ad libitum meal 3 hours after the test meal.

### 4.4. Biological Sample Collection

Repeated blood samples were obtained by inserting a venflon catheter into the antecubital vein of the right arm throughout each test day. Baseline samples were collected 20 min before the meals, as well as 20, 40, 60, 90, 120, 180 min afterwards (Figure 3b). The blood samples were transferred into 4 mL vials with heparin as an anticoagulant and centrifuged immediately at 3000× *g*, 4 °C for 10 min, and then aliquoted into 2 mL cryotubes and frozen at −80 °C until analysis. 

Participants had been asked to void morning urine into the toilet before arriving at the ward. Fasting urine samples were collected approx. 20 min before each meal upon arrival at the center as baseline samples from each participant. The rest of the urine samples were collected as complete samples within the following intervals: 0–1.5 h, 1.5–3 h, and 3–24 h. A 24 h pooled urine sample was produced by mixing aliquots of the three interval urine samples in relative proportion to their volumes (Figure 3b). Collected urine samples were stored below 5 °C in cooling bags during sampling until completion of each collection interval, after which aliquots were immediately transferred to 1 mL cryotubes and stored at −80 °C until analysis. Volunteers accepting long-term storage of their samples also donated a sample set for the CUBE biobank (Copenhagen University Biobank for experimental research, www.cube.ku.dk).

### 4.5. UPLC-QTOF-MS Analysis

Blood and urine samples were thawed and prepared for analysis as described previously [39,40]. Blood samples, urine samples, and additional pooled samples, used as quality control for each biofluid, were placed in 96-well microtiter plates (Waters, Hedehusene, Demark). The samples (urine or blood) from the same subject were kept in the same plate, while the sequence of samples was randomized within a plate; the subjects were randomized between plates (analytical batches) to minimize the influence of batch effects on within-person variations. The preparation of the plate followed the steps described previously [41]. All samples in plates were analyzed by UHPLC coupled to a quadrupole time-of-flight mass spectrometer (Premier QTOF, Waters Corporation, Manchester, UK) in full scan mode. Blanks and external metabolomic standard mixtures [42] were injected after every 30 samples, throughout each plate [42]. The injection volume was 5 µL. Details on chromatographic and mass-spectrometric conditions are reported in Supplementary Material S5. After the selection of putative seaweed intake biomarkers, further analysis was performed for biomarker structural characterization by a UHPLC system coupled to a Vion IMS QTOF mass spectrometer (Waters) and using the same chromatographic conditions as in the Premier QTOF.

Firstly, full scan acquisition was performed on selected urine samples for measuring the ions of interest at a higher accuracy. Secondly, data-dependent acquisition (DDA) was performed by including the ions of putative biomarkers for fragmentation and applying a mass-dependent ramp of collision-induced dissociations (CID) energies (from 10 eV to 30 eV for low *m/z* 50, and from 30 eV to 50 eV for high *m/z* 1,000). Finally, targeted MS/MS fragmentation experiments were performed on the selected biomarkers by using 14 eV, 28 eV, and 42 eV as CID energies.

### 4.6. Seaweed Extraction and Analysis

Seaweed extracts were produced to support the identification of seaweed originating metabolites. Two types of blended seaweed (UP and LD, 0.5 g amounts) were weighed out and extracted on the same day. UP and LD were extracted both in either methanol or ethanol (both 0.05 g/mL) and homogenized for 1 min. Ultrasound-assisted (115 V, 60 Hz intensity) extraction of the four homogenates was performed at 25 °C for 3 h with 6 breaks of 5 min each for cooling down and reducing oxidative degradation. The sample was precipitated by gravity, and each supernatant was collected into a new bottle. The extraction was repeated twice in order to obtain non-colored residues. The supernatants from each corresponding sample were combined, vacuum-centrifuged for 24 h, and the dry residues were stored in the freezer (−80 °C) until analysis by LC–MS. The method was modified to adjust to our lab condition based on the published paper [2].

Each sample was redissolved in 0.5 mL solvent A (0.1 % formic acid in Milli–Q water) and centrifuged for 10 min under 10,000× *g* (Eppendorf, Centrifuge 5417 R, Hamburg, Germany). The samples were analyzed by UHPLC coupled to Vion IMS QTOF (Waters), in the same analytical conditions as described for urine and blood. Urine samples were rerun in the same batch as the seaweed extracts for comparison.

### 4.7. Data Preprocessing

The raw acquired data were transferred into .netcdf (network Common Data Form) format data files by Databridge and imported into MZmine2 (ver. 2.31) [43]. The following steps were performed to generate the sets of arranged data from positive and negative mode: (1) peak detection for excluding noise; (2) chromatograph builder for shaping peaks; (3) chromatogram deconvolution (local minimum search) for removing the extra noise and making the peaks as similar as originals; (4) deisotoping for removing irrelevant isotopes; (5) peak alignment for combining the same peaks from all samples; (6) duplicate peak filter for discarding the repeated peaks; (7) peak list row filter for removing peaks only present in a few samples; (8) gap-filling (peak finder) for integrating baseline at the expected RT for any missing peaks. The detailed parameter settings for both modes are shown in Supplementary Material S6. The MZmine2-preprocessed feature intensity, RT, and *m/z* were transferred into MATLAB R2014b (ver. 8.4.0.150421) for data analysis. Feature pre-cleaning was conducted by removing irrelevant features [38,44] including those: (1) present in blank samples; (2) eluted too early (<0.3 min) or late (>9.5 min); (3) being potential isotopes or duplicate features; (4) not present in 70% of samples from each treatment and time. The Galaxy workflow4metabolomics [45] procedure (linear regression using sample pools) was used for batch correction to remove instrumental drift and any offset between batches.

### 4.8. Data Analysis

Data analysis was performed in MATLAB to find features differentiating between the treatments at each time point. The features were further selected as putative BFIs if the excretion profile showed an increase from baseline after UP or LD consumption but not after the control treatment.

In order to screen for features specific to seaweed (LD + UP) or to either LD or UP, n-way ANOVA [46] was employed by setting treatment number and time as fixed factors, thereby blocking parameters potentially accounting for the additional variance. The interaction term between the fixed factors was considered as well as the random effect (RE) from subjects. The 20 healthy people were randomized into the experiment, therefore they were not supposed to contribute so much as the fixed effect to variance. Note that urine samples and 24 h pooled urine samples were analyzed by n-way AVOVA separately. Therefore, we can identify which features are significantly different between meals as well as between time-points. Differences were considered statistically significant at *p* < 0.05 (without the use of false discovery rates) because this first screening is exploratory.

Multiple comparison tests [47] (post-hoc) followed by n-way ANOVA were applied for further exploring which features indeed were significant along time series using Dunn–Šidák corrections to contrast the control treatment with each of the other treatments (or with both) at each time point [48]. Only features specific to each meal or to both seaweed meals at several time-points were regarded as important for further study.

### 4.9. Standards and Identification

#### 4.9.1. Identification

The putative intake biomarkers selected by the statistical analysis were matched with an in-house database and different online databases such as HMDB [49], FOODB [50], Metlin [51], mzCloud (https://www.mzcloud.org/), SIRIUS (version 4.4.29) [52] and Chemspider [53] for precursor identification. Additionally, they were compared with previously identified seaweed biomarkers, as reported in our review [54].

Furthermore, samples analyzed in DDA mode were analyzed by the molecular networking method (MN [55,56] on the GNPS platform [30] in order to match both precursor ions and corresponding fragment ions with MS/MS spectral libraries. In addition, structure propagation of unknown compounds was performed based on the built network. Chemical structural annotation was further supported through in silico structure annotation with network annotation propagation (NAP) [57] and allocation-unsupervised substructure discovery (MS2LDA) [58] The MolNetEnhancer workflow [59] was used to summarize the obtained information within the mass spectral molecular network.

Finally, the putative intake biomarkers that did not match with any of the above databases, and those which did not result in any significant output from the GNPS platform were analyzed by targeted MS/MS. The acquired MS/MS spectra were manually investigated.

The putative intake biomarkers were classified as level I–V based on established MSI criteria [60]. In brief, level I was used when RT, *m/z*, and MS/MS spectra matched with a commercial standard; level II when MS/MS spectra matched with a compound reported in the literature or spectral libraries. This level was also deduced when no literature or spectral libraries were available, but only one possible structure fits the experimental information; level III was used when only the class of a target compound was identified; level IV was used when the formula of the compound was equivocal; level V when the compound was unknown.

#### 4.9.2. Standard

Loliolid was purchased from ChemFaces (Wuhan, China). Attempts to synthesize loliolid glucuronide was made as to the in-house protocol [41].

### 4.10. Deconjugation of the Urine Sample

To further support the identification of metabolites supposed to be conjugated to glucuronic acid, a deconjugation experiment was performed. In particular, 200 µL raw urine sample, 800 µL acetate buffer (0.1 M, pH 5.5), and 40 µL mixture of β-glucuronidase/arylsulfatase (obtained from Helix pomatia, CAS: 9001-45-0, Sigma) were mixed in an Eppendorf tube and stirred at 37 °C for 16 h. Ice-cold MeOH (40 µL) was subsequently added to stop the reaction. Then, the mixture was centrifuged at 10,000× *g* for 5 min. The supernatant was collected and analyzed by UHPLC coupled to Vion IMS QTOF with the same full scan method as described in Section 4.5.

## 5. Conclusions

In conclusion, we were able to discover four metabolites that are increased following recent brown seaweed consumption. Loliolid glucuronide (M3) and isololiolid glucuronide (M2) are phase II metabolites of compounds originating from the seaweeds. One of the other two compounds (M4) has been tentatively classified as a glycoside or a carbohydrate (level IV), while compound M1 was tentatively identified as hydroxy-dihydrocoumarin at level III. M1 and M4 still need to be further structurally characterized. However, the four putative BFIs may not be unique to brown seaweed intake, requiring further validation. Further validation and investigation of the combination of the four markers may be needed to develop BFIs for studying seaweed intake and the health effects of seaweeds in humans.

## Figures and Tables

**Table 1:** Urine metabolites originating from seaweed and/or biotransformation of seaweed components in both positive and negative modes. The retention time (RT) and the *m/z* reported here have been measured with a UHPLC system coupled to a Vion IMS QTOF mass spectrometer (Waters).

No.	Polarity	Experimental *m/z*	Theoretical Monoisotopic Mass	RT	MS/MS	Molecular Formula	Annotation	Suggested Metabolite _a_
M1	POS	137.0596	136.0524	4.73	109.0645 81.06939 55.01729	C_8_H_8_O_2_	unknown fragment	hydroxyl-dihydrocoumarin-like ^III^
M2	POS	197.1185	196.1099	5.31	179.1069 161.0959 135.1170 133.1009 107.0854 105.0699 93.0697 91.0544	C_11_H_16_O_3_	[M-glucuronic acid + H_2_O + H]^+^	isololiolid glucuronide ^II^
	NEG	371.1347	372.1419	5.31	177.089, 113.023, 85.028, 75.0076	C_17_H_24_O_9_	[M–H]^-^	
M3	POS	197.1176	196.1099	5.37	179.1069 161.0959 135.1170 133.1009 107.0854 105.0699 93.0697 91.0544	C_11_H_16_O_3_	[M-glucuronic acid + H_2_O + H]^+^	Loliolid glucuronide ^I^
		373.1497	372.1419	5.37	197.1176179.1069 161.0959 135.1165 133.1010 107.0852 105.0697 93.0695 91.05389 84.04425	C_17_H_24_O_9_	[M + H]^+^	
	NEG	371.1346	372.1419	5.37	195.9997 177.0911 113.0232 85.02834 75.00761	C_17_H_24_O_9_	[M–H]^-^	
M4	NEG	467.2272	468.2359	6.14	193.0345 113.0236 85.0287 75.0080	C_24_H_36_O_9_	[M–H]^-^	Glycoside ^IV^

_a_ Identification level based on established MSI criteria [60].

## Supplementary Materials

The following are available online at https://www.mdpi.com/2218-1989/11/1/11/s1. S1. Results of n-way ANOVA and paired multiple comparisons. S1.1. n-way ANOVA results for random and fixed factors of seven selected significant features/ions. S1.2. Results of paired multiple comparisons for selected features/ions. S2. MS/MS spectra of M1–M4 metabolites. S2.1 Comparisions of MS/MS spectra of: (a) M1 (up) with 4-hydroxycoumarin (down); (b) M1 (up) with 7-hydroxycoumarin (down) obtained from mzCloud. MS/MS spectra of 4-hydroxycoumarin and 7-hydroxycoumarin were obtained from the MoNA database. The collision-induced dissociation energy applied to M1 was 14 eV. MS/MS spectra of the remaining compounds in both (a) and (b) are their average spectra. S2.2 Comparisions of MS/MS spectra of: (a) a commercial standard of loliolid (up)(a) with both glucuronidase treated M3 (down) in positive mode; (b) a commercial standard of loliolid (up) and with glucuronidase treated M2 (down) in positive mode; c1) synthesized loliolid glucuronide (up) with M3 (down) in negative mode; (c2) synthesized loliolid glucuronide (up) with M3 (down) in positive mode. The collision-induced dissociation energy applied to the compounds in both (a) and (b) was 14 eV and to the compounds in (c1) and (c2) was 28 eV. S2.3 Comparisions of MS/MS spectra of: (a) M4 (up) with glucuronic acid (down); (b) cyclodextrin obtained from mzCloud library. The collision-induced dissociation energy applied to M4 was set to 42 eV. The MS/MS spectra of glucuronic acid and cyclodextrin are their average spectra. S3. Comparison of extracted ion chromatograms (mass tolerance < 5 ppm) of (a) *m/z* 165.054 and (b) *m/z* 137.0596, and their relative MS/MS spectra (a1,b1) acquired with a dissociation energy of 14 eV, obtained from a urine sample in positive mode, illustrating that *m/z* 165.054 and *m/z* 137.0596 are likely belonging to the same seaweed compound. S4. Comparison of the MS/MS spectra of M2 (black color) acquired by random collision energy from the urine sample and loliolid (green color) from the GNPS library. The match was classified as a bronze-level with the cosine value 0.76 with seven matched peaks. S5. Analytical conditions of UHPLC-QTOF-MS. S5.1. Mobile phases and gradient. S5.2. Parameters of mass spectrometry. S6. The parameters of data preprocessing in MZmine2.
