# Supplementary material for: Discovery of Urinary Biomarkers of Seaweed Intake Using Untargeted LC–MS Metabolomics in a Three-Way Cross-Over Human Study
Source: Metabolites. 2020 Dec 28;11(1):11. doi: 10.3390/metabo11010011 (PMC7823344; doi:10.3390/metabo11010011)
Supplement: Supplementary file 1 [file metabolites-11-00011-s001.pdf]

S1. Results of n-way ANOVA and paired multiple comparisons

S1.1. n-way ANOVA results for random and fixed factors of seven selected significant features/ions

NO.	Mode	m/z	RT	*p-value* (RE)	*p-value* (Meal effect)	*p-value* (TP effect)	*p-value* (Meal effect at 24hr)
M1	POS	137.0596	4.73	0.0689	2.53E-05	7.24E-05	2.18e-05
M2	POS	197.1185	5.31	0.7806	2.0E-03	3.65E-09	1.53E-02
M2	NEG	371.1346	5.31	0.4798	2.0E-04	3.25E-13	4.18e-05
M3	POS	197.1176	5.37	0.5409	3.3E-03	2.29E-12	7.1E-03
M3	POS	373.1497	5.37	0.7877	1.1E-03	6.48E-09	2.32e-04
M3	NEG	371.1347	5.37	0.2598	8.0E-04	6.42E-09	2.6E-02
M4	NEG	467.2272	6.14	0.3513	3.0E-04	4.88E-12	2.16E-02

Abbreviation: RE: random effect, TP: time point

S1.2. Results of paired multiple comparisons for selected features/ions

m/z	RT	*p-value* LD vs control (3 hrs)	*p-value* UP vs control (3 hrs)	*p-value* 0 vs 1.5 hrs (control)	*p-value* 1.5 vs 3 hrs (control)	*p-value* 3 vs 24 hrs (control)
137.0596	4.6299	9.19E-01	4.98E-13	0.2063	1.0000	1.0000
197.1201	5.3480	3.70E-03	1.15E-06	1.0000	1.0000	1.0000
197.1230	5.2886	0.0000	2.00E-06	1.0000	0.9586	1.0000
373.1505	5.3483	4.7E-03	2.80E-07	1.0000	0.9999	1.0000
371.1149	5.3560	3.61E-05	1.39E-06	0.9998	1	1
371.1296	5.2886	2.76E-08	9.00E-08	1	0.9991	1
467.2272	6.0109	8.7E-03	1.14E-08	1	1	1

Abbreviation: TP: time point. Ps. TP1 means time point 1

S2. MS/MS spectra of M1-M4 metabolites.

S2.1 Comparisions of MS/MS spectra of: a) M1 (up) with 4-hydroxycoumarin (down) , b) M1 (up) with 7-hydroxycoumarin(down). . MS/MS spectra of 4-hydroxycoumarin and 7-hydroxycoumarin were obtained from the MoNA database. The collision-induced dissociation energy applied to M1 was 14 eV. MS/MS spectra of rest compounds in both (a) and (b) are their average spectra.

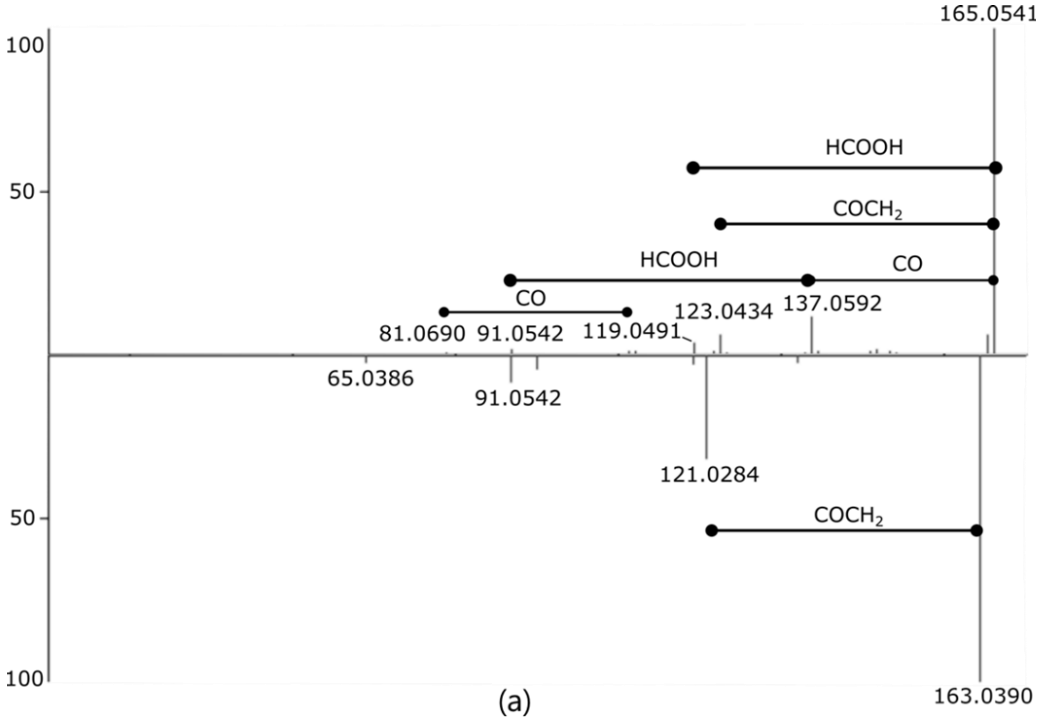

(a)

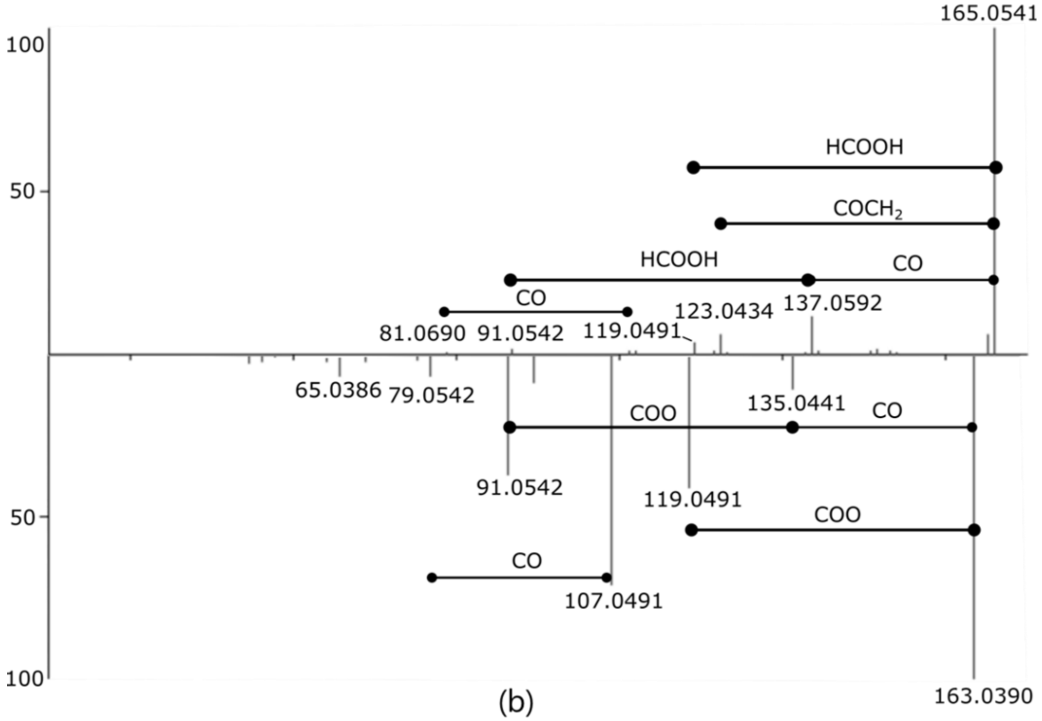

(b)

S2.2 Comparisions of MS/MS spectra of: a) a commercial standard of loliolid (up) with glucuronidase treated M3 (down) in positive mode, b) a commercial standard of loliolid (up) with glucuronidase treated M2 (down) in positive mode, c1) synthesized loliolide glucuronide (up) with M3 (down) in negative mode, c2) synthesized loliolide glucuronide (up) with M3 (down) in positive mode. The collision-induced dissociation energy applied to the compounds in both (a) and (b) was 14 eV and to the compounds in (c1) and (c2) was 28 eV.

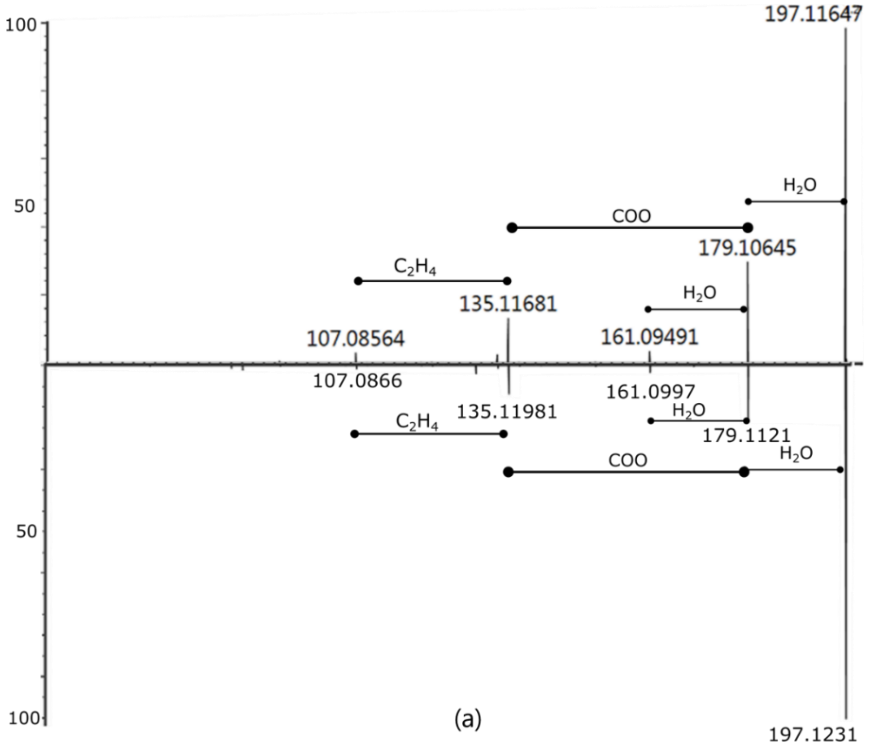

(a)

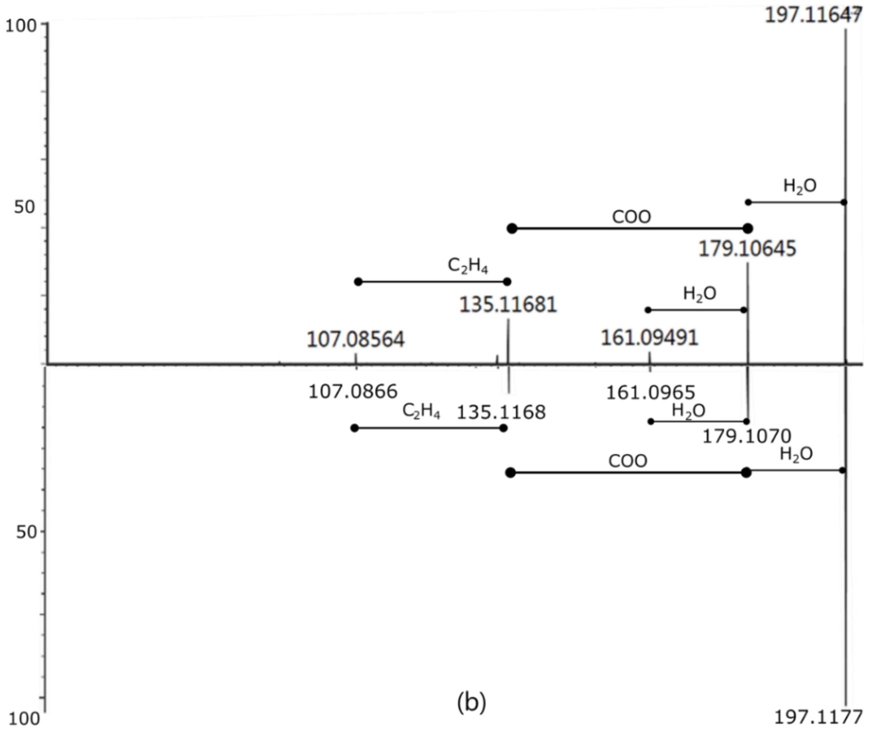

(b)

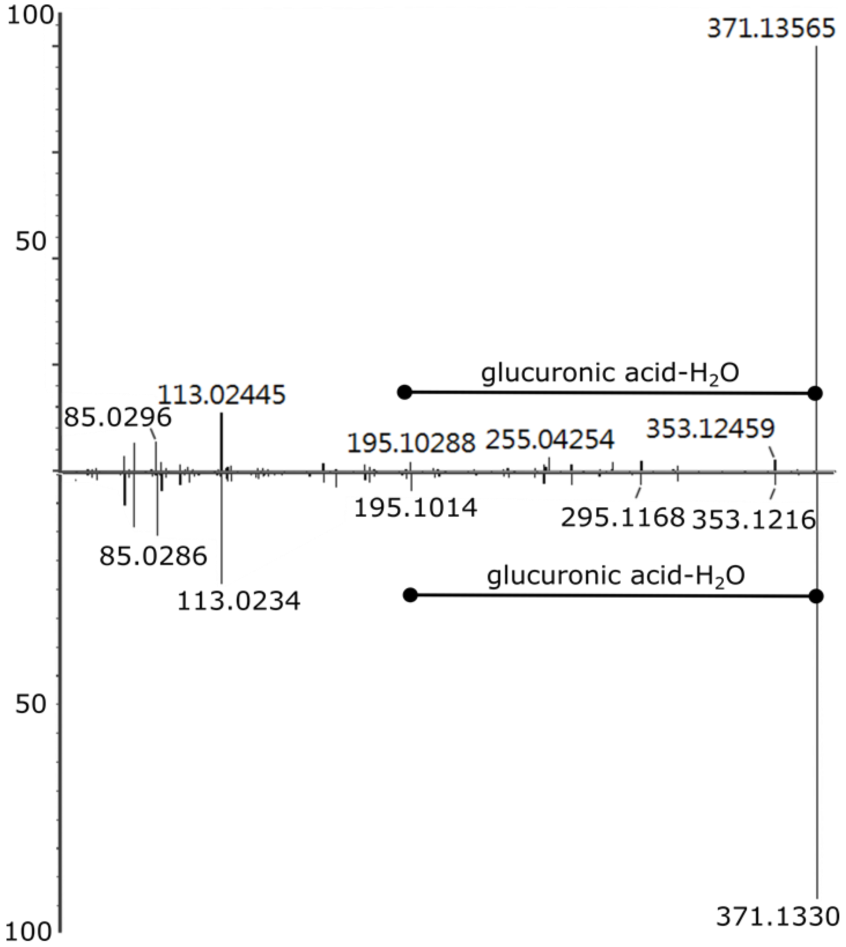
100
50
50
100
371.13565
glucuronic acid-H_2O
113.02445
85.0296
195.10288
255.04254
353.12459
195.1014
295.1168
353.1216
85.0286
113.0234
glucuronic acid-H_2O
371.1330

(c1)

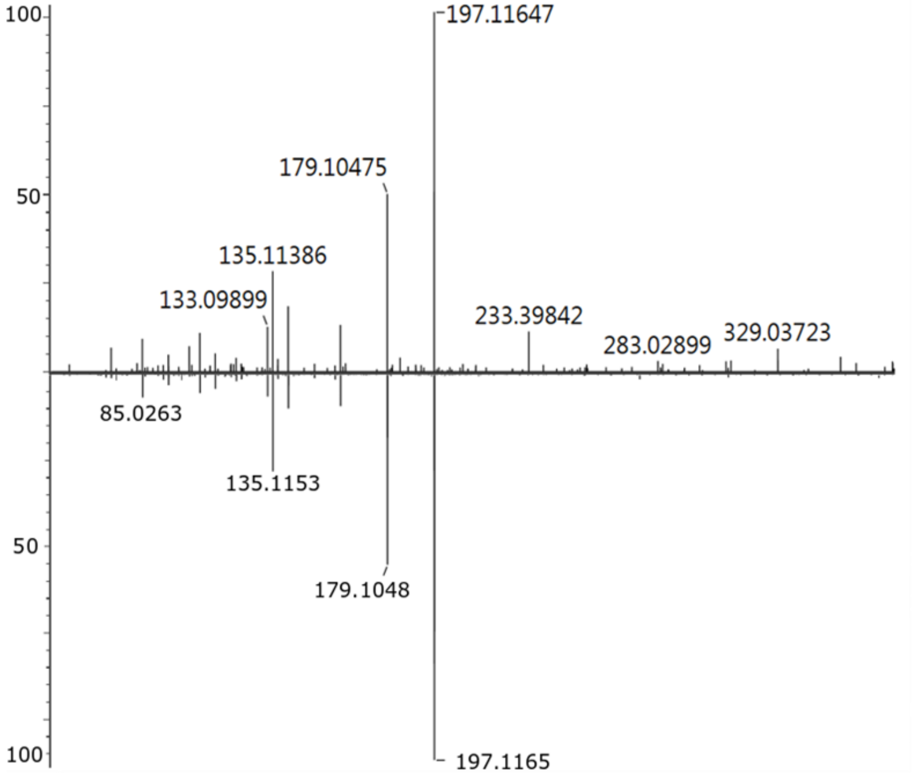
100
50
50
100
197.11647
179.10475
135.11386
133.09899
233.39842
283.02899
329.03723
85.0263
135.1153
179.1048
197.1165

(c2)

S2.3 Comparisions of MS/MS spectra of: a) M4 (up) with glucuronic acid (down) ,b) cyclodextrin obtained from mzCloud library. The collision-induced dissociation energy to applied to M4 was set to 42 eV. The MS/MS spectra of glucuronic acid and cyclodextrin are their average spectra.

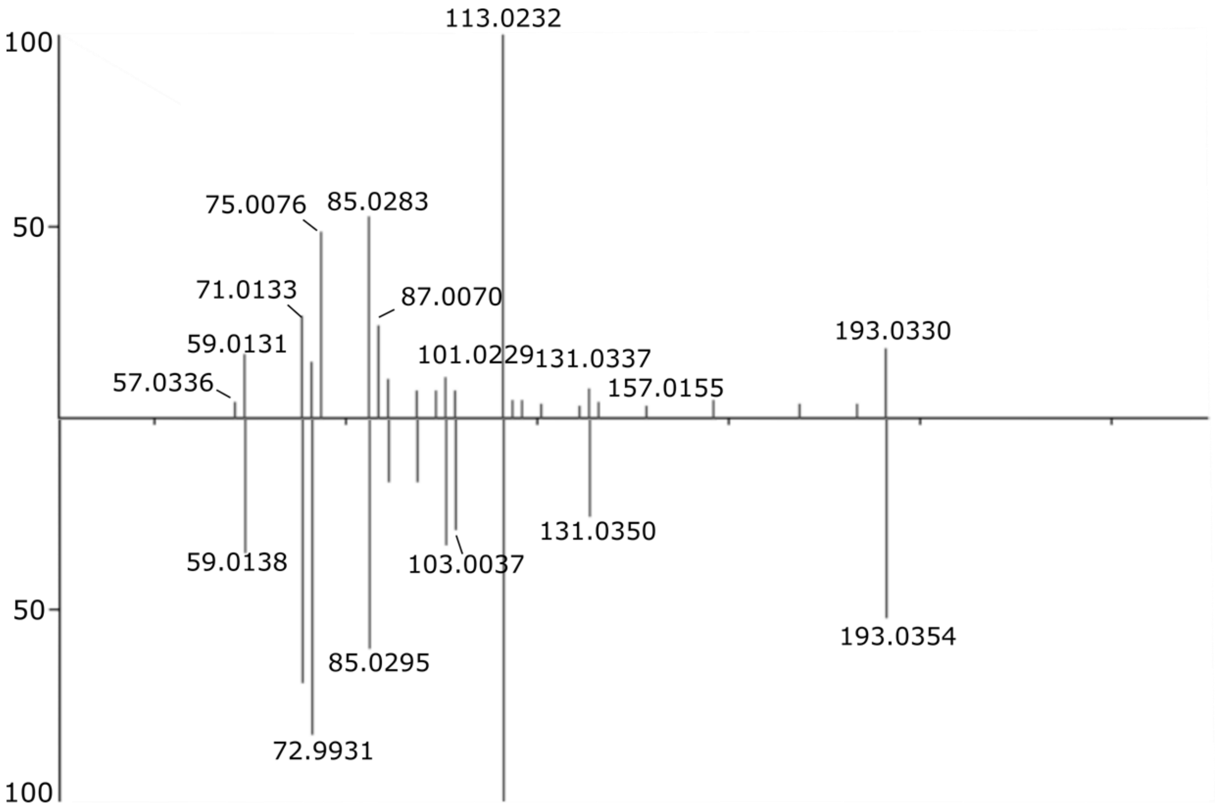

(a)

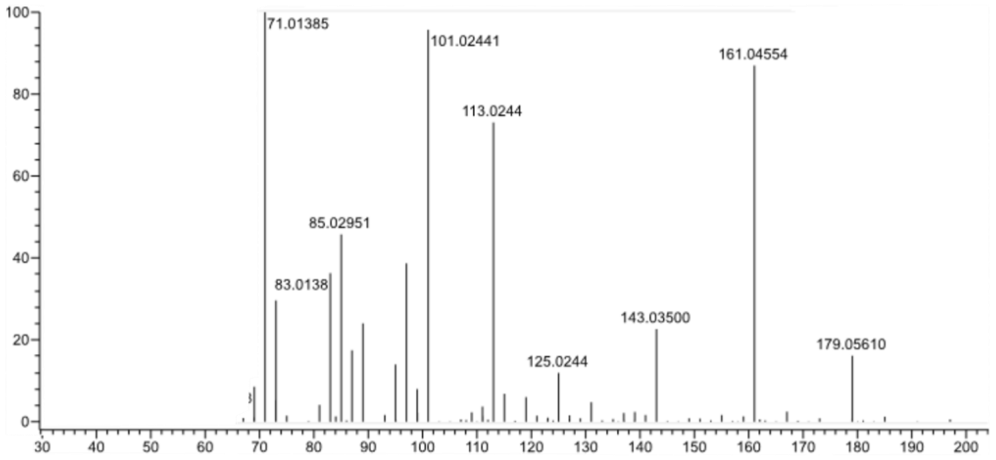

(b)

S3. Comparison of extracted ion chromatograms (mass tolerance < 5 ppm) of a) *m/z* 165.054 and b) *m/z* 137.0596, and their relative MS/MS spectra (a1, b1) acquired with a dissociation

energy of 14 eV, obtained from a urine sample in positive mode, illustrating that *m/z* 165.054 and *m/z* 137.0596 are likely belonging to the same seaweed compound.

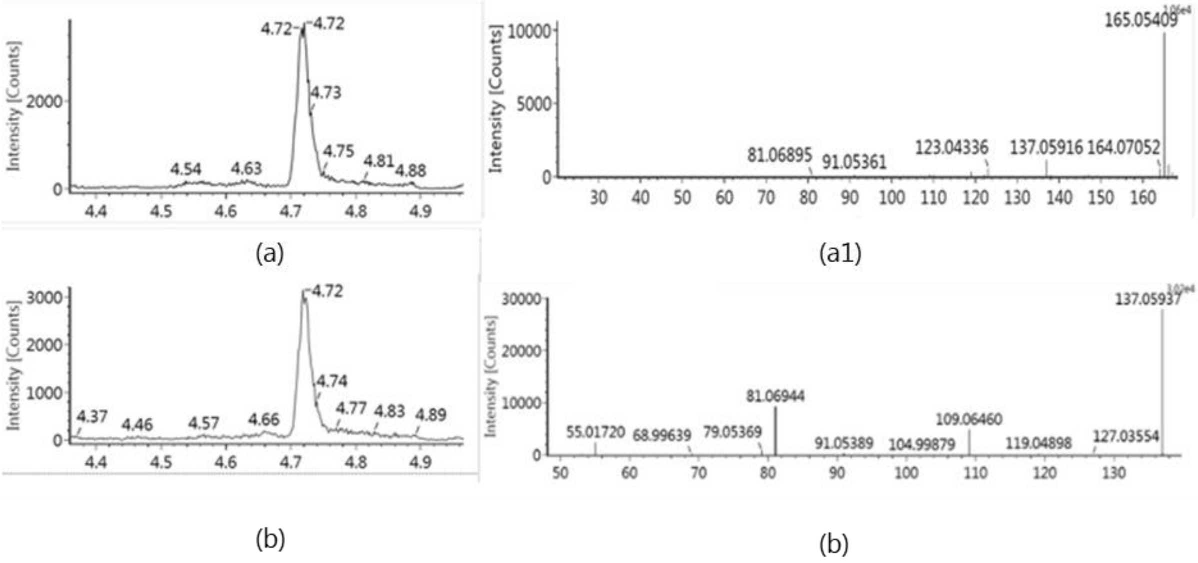

S4. Comparison of the MS/MS spectra of M2 (black color) acquired by random collision energy from the urine sample and loliolid (green color) from the GNPS library. The match was classified as a bronze-level with the cosine value 0.76 with seven matched peaks.

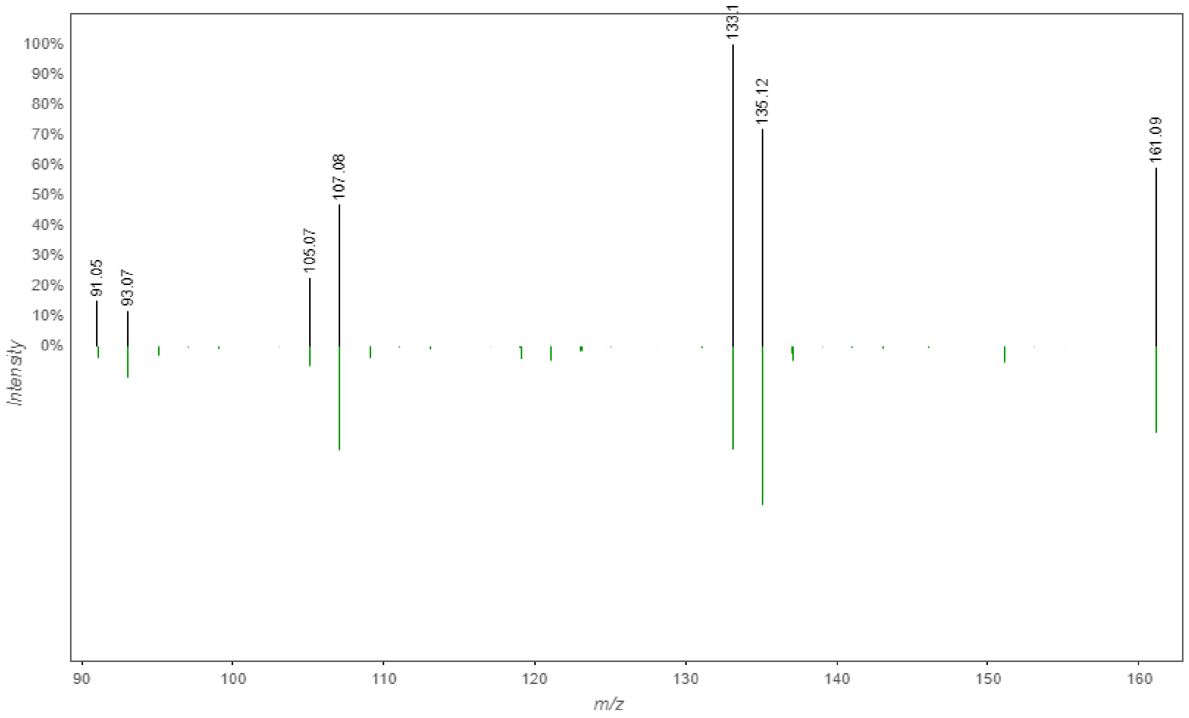

S5. Analytical conditions of UHPLC-QTOF-MS

S5.1 Mobile phases and gradient

The mobile phases were A (0.1% HCOOH in water), B (methanol), C (100mM CH_3COONH_4 in methanol), and D (isopropanol). The chromatographic separation was performed at a flow rate of 0.4 mL/min using a 2.1 mm ø × 100 mm HSS T3 column with 100 Å pore size and 1.8 μm particle size (Milford, USA) coupled with an HSS T3 C18 column (2.1x5 mm, 1.8 μm) as pre-column at a temperature of 50 ℃. The gradient is shown in the table below. The same gradient was used for both modes.

Time (min)	Flow Rate (mL min^{-1})	Mobile phase A (%)	Mobile phase B (%)	Mobile phase C (%)	Mobile phase D (%)	Curve
0.00	0.4	100	0	0	0	Initial
0.75	0.4	100	0	0	0	6
6.00	0.5	0	100	0	0	7
6.50	0.5	0	0	70	30	7
8.00	0.6	0	0	70	30	7
8.10	0.4	0	0	70	30	6
9.00	0.4	100	0	0	0	5
10.00	0.4	100	0	0	0	5

S5.2 Parameters of mass spectrometry

The samples were acquired in both positive (+) and negative (-) polarity modes. The capillary voltage was set as 3.2 kV (+) and 2.8 kV (-) and the cone voltage was 20kV. The cone gas and desolvation gas flow were set to 50 L hr^{-1} and 1000 L hr^{-1}, respectively. The temperature of the ion source and desolvation nitrogen gas was set to 120 °C and 400 °C, respectively. Leucine-enkephalin (100 pg mL^{-1} in 0.1% HCOOH in H_2O: ACN 50:50, v/v) was used as a lock-spray agent to correct the mass accuracy along with the data acquisition every 1 min. Masses were generated in the real-time range from 50 to 1500 *m/z*.

S6. The parameters of data preprocessing in MZmine2

Steps	Parameters	Urine_pos	Urine_neg	Serum_pos	Serum_neg

Mass detection	Noise level	1.0E1	1.0E1	1.0E1	1.0E1
Chromatograph builder	Min time span (min)	0.04	0.038	0.041	0.038
	m/z tolerance	0.01	0.0095	0.0117	0.0112
	Min height	50	50	50	50
Chromatogram deonvolution (local minimum search)	Chromatographic threshold	97%	98%	99%	99%
	Search minimum RT range (min)	0.04	0.02	0.03	0.03
	Minimum relative height	3.29%	3.28	3.29%	3.29%
	Minimum absolute height	75	40	53	53
	Min ratio of peak top/edge	1.0	1.0	1.0	1.0
	Peak duration range(min)	0.01-0.2	0.007-0.2	0.001-0.2	0.01-0.2
Deisotoping (isotopic peaks grouper)	m/z tolerance	0.06	0.06	0.06	0.06
	RT tolerance	0.01	0.01	0.01	0.01
Peak alignment (join aligner)	m/z tolerance	0.0143	0.0083	0.0114	0.01
	RT tolerance	0.14	0.06	0.08	0.1
Duplicate peak filte	m/z tolerance	0.01	0.01	0.01	0.01
	RT tolerance	0.01	0.01	0.01	0.01
Peak list row filter	Minimum peaks in a row	16	16	16	16
Gap filling (peak finder)	Intensity tolerance	1%	1%	1%	1%
	m/z tolerance	0.01	0.0095	0.01	0.01
	RT tolerance	0.04	0.038	0.04	0.04

Abbreviation: m/z: mass to charge ratio; RT: retention time; Intensity: peak height
